# Assessing antimicrobial resistance in *Campylobacter jejuni* and *Campylobacter coli* and its association with antimicrobial use in Canadian turkey flocks

**DOI:** 10.1017/S0950268823001462

**Published:** 2023-09-05

**Authors:** Rima D. Shrestha, Agnes Agunos, Sheryl P. Gow, Csaba Varga

**Affiliations:** 1 Department of Pathobiology, College of Veterinary Medicine, University of Illinois Urbana-Champaign, Urbana, IL, USA; 2 Department of Internal Medicine, University of Illinois College of Medicine Peoria, Peoria, IL, USA; 3 Foodborne Disease and Antimicrobial Resistance Surveillance Division, Center for Foodborne, Environmental and Zoonotic Infectious Diseases, Public Health Agency of Canada, Guelph, ON, Canada; 4 Center for Foodborne, Environmental and Zoonotic Infectious Diseases, Public Health Agency of Canada, Saskatoon, SK, Canada; 5 Carl R. Woese Institute for Genomic Biology, University of Illinois Urbana-Champaign, Urbana, IL, USA

**Keywords:** antimicrobial resistance, antimicrobial use, *Campylobacter coli*, *Campylobacter jejuni*, Canada, fluoroquinolone, multidrug resistance, tetracycline, turkey

## Abstract

Turkeys are important sources of antimicrobial-resistant Campylobacter. A total of 1063 isolates were obtained from 293 turkey flocks across Canada between 2016 and 2021 to evaluate their antimicrobial resistance (AMR) prevalence, patterns, distribution, and association with antimicrobial use (AMU). A high proportion of *C. jejuni* and *C. coli* isolates were resistant to tetracyclines and fluoroquinolones, despite the very low use of these drugs. *C. jejuni* isolates had a higher probability of being resistant to tetracyclines than *C. coli* isolates. The chance of *C. jejuni* isolates being resistant to fluoroquinolones, macrolides, and lincosamides was lower compared to *C. coli.* Isolates from the western region had a higher probability of being resistant to fluoroquinolones than isolates from Ontario. Isolates from Ontario had higher odds of being resistant to tetracyclines than isolates from Quebec. No associations were noted between the resistance and use of the same antimicrobial, but the use of certain antimicrobial classes may have played a role in the maintenance of resistance in Campylobacter (fluoroquinolone resistance – bacitracin and streptogramin use, tetracycline resistance – flavophospholipids and streptogramins use, macrolide resistance – flavophospholipid use). Low-level multidrug-resistant Campylobacter was observed indicating a stable AMR in turkeys. This study provided insights aiding future AMU and AMR surveillance.

## Introduction

*Campylobacter* is the major source of foodborne enteric infections in North America and worldwide [1, 2]. Mainly *C. jejuni* (~90% of cases) and occasionally *C. coli* (5–10% cases) are linked with human campylobacteriosis [1–3]. In the United States of America, 20 campylobacteriosis cases per 100,000 population are reported annually [3], while in Canada, the adjusted rate of campylobacteriosis cases was estimated to be 447 per 100,000 person-years [2]. Campylobacteriosis in humans is mainly sporadic, clinically mild, and self-limiting [1]; however, in some instances, *Campylobacter* infections can be severe and require treatment with antimicrobials in young, elderly, and immunocompromised people. Post-infection sequelae of campylobacteriosis, such as irritable bowel syndrome, Guillain–Barré syndrome, and reactive arthritis, also increase its health burden [1, 3].

The World Health Organization (WHO) listed *Campylobacter* spp. among the top six highly prioritized antimicrobial-resistant pathogens because it became increasingly resistant to clinically important antimicrobials in human medicine [4]. Identifying the sources of resistant *Campylobacter* isolates occurring in animals, humans, and their environment and identifying risk factors that impact the emergence of antimicrobial resistance (AMR) is warranted.

Poultry and poultry products are the main sources of *Campylobacter* infection in people [5]. Poultry shed *Campylobacter* in their faeces, which can be transmitted to humans through direct contact with infected poultry or indirect exposure to a contaminated environment, food, or water. A previous study from Canada has shown that tetracycline-resistant *Campylobacter* isolates from poultry meat were genetically linked to human cases [6]. Additionally, a high prevalence of fluoroquinolone- and tetracycline-resistant *Campylobacter* isolates of poultry origin, including turkey flocks and meat, has been reported in Europe [7–9], Asia [9, 10] Australia [11], and North America [6, 12–16].

Antimicrobials are commonly used in turkeys at the flock level in Canada to prevent and treat bacterial infections [17] as with any other poultry and livestock species [18]. However, it has been demonstrated that the use of antimicrobials in turkey flocks can impact the emergence and maintenance of antimicrobial-resistant *Campylobacter* within the flock [19] and that resistance can persist in the environment when biosecurity is not optimal [20]. As Canada is among the world’s top turkey producers and because turkey meat is an important component of the Canadian food supply, AMR and antimicrobial use (AMU) in turkey farms have been monitored through the Canadian Integrated Program for Antimicrobial Resistance Surveillance (CIPARS) since 2013 [14]. The Canadian turkey industry developed an AMU reduction strategy to limit the emergence of AMR in their sector [21]. The elimination of the preventive use of medically important antimicrobials and the reduction of the total amount of antimicrobials used on turkey farms has shown a promising effect in reducing the selection of drug-resistant *E. coli* isolates [21].

To better understand AMR in *Campylobacter* isolated on Canadian turkey farms and AMU factors impacting AMR, this study utilized the CIPARS data collected from 2016–2021 to (i) estimate the prevalence of resistance in *Campylobacter* isolates, (ii) evaluate the AMR patterns and differences in AMR among different *Campylobacter* species, and (iii) evaluate associations between AMR and AMU. This study will help inform refinements of the industry-led AMU reduction strategy and identify areas for further research.

## Methods

CIPARS implements on-farm surveillance of commensal and foodborne pathogens, including *Campylobacter* spp. Since 2013 surveillance has included susceptibility testing of *Campylobacter* spp. isolates and the collection of AMU data in turkey flocks. However, for consistent and comparable data that included all major turkey-producing provinces (Ontario, Québec and the Western region (Alberta, Saskatchewan, and British Columbia)) only the 2016–2021 turkey AMR and AMU surveillance information were utilized for this study. A detailed description of the surveillance framework, and sampling plan of turkey flocks, including faecal sample and flock selection criteria, along with laboratory procedures is available in Supplementary Figure S1 and Supplementary Table S1. This information is also available from previous studies [22, 23].

### Faecal sample and AMU data collection

Briefly, poultry veterinarians collected sentinel farm data once a year, including AMU, after receiving informed consent from participating turkey producers. Veterinarians selected one flock per farm per year and collected one pooled (10 droppings) faecal sample per quadrant of the barn per flock during their yearly farm visit on the last week of the turkey’s growth period based on the intended market weight category. These faecal samples were submitted to the National Microbiology Laboratory (NML), Public Health Agency of Canada for *Campylobacter* isolation, typing, and antimicrobial susceptibility testing.

### Laboratory procedures

Briefly, 25 g of pooled faecal samples were added to 225 mL buffered peptone water, then 50 mL of this peptone rinse was mixed with 50 mL of double-strength Bolton broth, and the broth was incubated at 42 ± 1°C for 44–48 h. A loopful of broth was then streaked onto a modified Charcoal Cefoperazone Deoxycholate Agar (mCCDA) plate and incubated in a microaerobic atmosphere at 42 ± 1°C for 24–72 h. Suspect colonies were streaked again onto a new mCCDA plate, and incubated, one colony from this plate was streaked onto a Mueller Hinton (MH) blood agar plate and incubated at 42 ± 1°C for 24–48 h. All incubation steps were performed in a microaerobic atmosphere. A colony from the MH plate was tested for catalase, oxidase, and Gram stained to determine the presumptive *Campylobacter* colonies. Multiplex PCR (mPCR) [24] was used to speciate the presumptive *Campylobacter* to ‘*C. jejuni*’ or ‘*C. coli*’. Those unidentified *Campylobacters* (no specific primer sets used for less-frequently occurring species) were referred to as ‘*Campylobacter* spp.’

One *Campylobacter-*positive colony from each faecal sample was incubated on an MH plate at 42 ± 1°C for 24 h, and the colony from the incubated MH plate was mixed with horse blood before being dispensed onto a CAMPY plate designed by the National Antimicrobial Resistance Monitoring System (NARMS) of the United States of America for testing using a broth microdilution method (Sensititre system (Trek Diagnostic Systems Ltd, West Sussex, England) according to Clinical and Laboratory Standards Institute (CLSI) (M45-A2) to obtain the Minimum Inhibitory Concentration (MIC) values for *Campylobacter.* The CAMPY plate contained nine antimicrobials (ciprofloxacin, telithromycin, azithromycin, clindamycin, erythromycin, gentamicin, nalidixic acid, florfenicol, and tetracycline). *C. jejuni* ATCC 33560 was used for quality control.

Based on the CLSI breakpoints available, MIC values for each isolate were interpreted as ‘susceptible’, ‘intermediate’, and ‘resistant’. Following the NARMS interpretative criteria and the European Committee on Antimicrobial Susceptibility Testing (EUCAST) Steering Committee guidelines studies, isolates showing the ‘intermediate’ MIC values were classified as ‘susceptible’ for data analysis [25, 26].

### Data management and analysis

Responses to the farm questionnaires were entered into a PostgreSQL database customized for CIPARS and extracted in Microsoft Excel (Microsoft 365) format. These data were merged with laboratory data on AMR using unique flock identification variables, veterinary practice, and sample date before statistical analyses were performed in R software (R core team 2022) in the R studio platform (R studio, PBC, 2022). All data manipulation, descriptive and cluster analyses were performed in R software using ggplot2, RColorBrewer, and R base packages. All logistic regression models were constructed using a generalized estimating equation (GEE) with an exchangeable correlation structure that accounted for clustering at the flock level using Stata v18 (Stata Corp., College Station, TX).

For each isolate, resistance was summarized at the antimicrobial class level (Supplementary Table S2). The isolate was considered multidrug-resistant (MDR) if it exhibited resistance to ≥3 antimicrobial classes. The AMU indicator used was the mg/kg turkey biomass, estimated at the flock level for total AMU and class-specific use (Supplementary Table S2).

### Descriptive statistics

Frequencies, percentages, and proportions were determined for categorical variables. For continuous variables, mean, median and interquartile range were determined. The proportion of resistance to each antimicrobial class was determined by dividing the number of resistant isolates to that antimicrobial class by the total number of isolates tested for that antimicrobial class. The Clopper–Pearson method was used to estimate the exact binomial confidence intervals (95% CI) for proportions.

### AMR pattern clustering

Ward’s hierarchical single-linkage clustering with the Euclidean distances method was applied [27] to construct heatmap dendrograms to compare the similarity in the class-level AMR patterns among *Campylobacter* species and sampling regions.

### Logistic regression analysis

Population-averaged univariable and/or multivariable logistic regression models were fitted using GEEs, with an exchangeable correlation structure that accounted for clustering at the flock level. Only variables with enough variability (≥5% or ≤95%) were included.

The model building consisted of two steps: univariable and multivariable, respectively. The predictor variables with *p* < 0.20 in the univariable stage were included in the multivariable model. Manual stepwise-backward elimination was performed, and the final model included predictors with a statistically significant association (*p* ≤ 0.05) on the Wald *χ*2 test. The odds ratio (OR), 95% CIs, and *p*-values were reported for all the model outcomes. Predicted probabilities for AMR were calculated from each model and displayed graphically.

#### *Assessing differences in AMR among* Campylobacter *species*

The outcome variables represented the resistance status of isolates to each antimicrobial class. *Campylobacter* species/group were included as predictor variables, selecting *C. coli* as the referent category to which *C. jejuni* and *C.* spp. were compared).

#### Assessing differences in AMR among Canadian regions

The outcome variables represented the resistance status of *Campylobacter* isolates (irrespective of species) to each antimicrobial class. Canadian regions were included as predictor variables, selecting Ontario as the referent category to which Quebec and Western regions were compared.

#### Assessing association between AMR and AMU

The outcome variable represented the resistance status of *Campylobacter* isolates (irrespective of species) to each antimicrobial class. The predictor variables included flock-level AMU variables.

## Results

### Campylobacter recovery and speciation

Overall, 1063 *Campylobacter* were isolated from 293 Canadian turkey flocks monitored between 2016 and 2021. Of these, 337 isolates were from 93 flocks in Ontario, 223 isolates were from 62 flocks in Québec, and 503 isolates were from 138 flocks in Western Canada. Four *Campylobacter* isolates each were recovered from 228 flocks, three isolates each from 35 flocks, two isolates each from 16 flocks, and one isolate from 14 flocks. Speciation of the 1063 isolates were identified as *C. jejuni* (*n* = 651; 61.24%), *C. coli* (*n* = 336; 31.61%), and the remaining isolates were unidentified *Campylobacter* species (*n* = 76; 7.15%). The highest number of turkey farms enrolled and sampled was in 2021, which is also the year with the highest number of *Campylobacter* isolates recovered (Supplementary Table S3).

### Analysis of AMR percentages and minimum inhibitory concentrations

Of the 1063 *Campylobacter* isolates, 61.99% (*n* = 659) were resistant to at least one antimicrobial. The number of isolates that were resistant to at least one antimicrobial throughout the study period is illustrated in Supplementary Figure S2. The highest number of isolates showed resistance to tetracycline and quinolones between 2016 and 2021.

The distribution of MIC values for the antimicrobials examined (*n* = 9) is summarized in Supplementary Table S4. The prevalence of AMR in *Campylobacter* at the isolate and species level is presented in Table 1 *Campylobacter* isolates had a high flock-level prevalence of resistance to tetracycline (*n* = 458; 43.13%) and fluoroquinolones (*n* = 300; 28.22%), low prevalence of resistance to macrolides (*n* = 91; 8.56%) and lincosamides (*n* = 61; 5.74%), and very low prevalence of resistance to ketolides (*n* = 4; 0.38%). All isolates were susceptible to aminoglycosides and phenicols. Higher resistance to tetracycline was found in *C. jejuni,* as compared to *C. coli* isolates (Table 1). Resistance to fluoroquinolones, macrolides, and lincosamides was highest in *C. coli,* followed by *C.* spp. isolates. Ketolide resistance was found only in *C. coli* and *Campylobacter* spp. isolates.

**Table 1.** Prevalence of antimicrobial resistance in *Campylobacter* at isolate and species levels

Resistance to antimicrobial class^a^	*Campylobacter* (*n* = 1338) % [95% CI]^b^	*Campylobacter jejuni* (*n* = 871) % [95% CI]^b^	*Campylobacter coli* (*n* = 383) % [95% CI]^b^	*C.* spp.^c^ (*n* = 84) % [95% CI]^b^
Aminoglycosides	0 (0.00–0.45)	0 (0–0.73)	0 (0–1.41)	0 (0–6.0)
Ketolides	0.38 (0.12–1.03)	0 (0–0.73)	0.6 (0.1–2.37)	2.63 (0.46–10.05)
Lincosamides	5.74 (4.45–7.35)	0.61 (0.2–1.68)	14.58 (11.08–18.92)	10.53 (4.98–20.21)
Macrolides	8.56 (6.98–10.45)	0.77 (0.28–1.89)	22.02 (17.79–26.91)	15.79 (8.77–26.35)
Phenicols	0 (0.00–0.45)	0 (0.00–0.73)	0 (0.00–1.41)	0.00 (0–6.0)
Quinolones	28.22 (25.55–31.05)	22.43 (19.32–25.87)	38.1 (32.92–43.55)	34.21 (23.96–46.07)
Tetracyclines	43.09 (40.09–46.13)	49.16 (45.25–53.07)	33.63 (28.65–38.99)	32.89 (22.8–44.73)

a Antimicrobial susceptibility testing was conducted for these antimicrobials: Aminoglycosides (Gentamicin), Ketolides (Telithromycin), Lincosamides (Clindamycin), Macrolides (Azithromycin and Erythromycin), Phenicols (Florfenicol), Quinolones (Ciprofloxacin and Nalidixic acid), and Tetracyclines (Tetracycline).

b The exact binomial 95% confidence interval was calculated.

c Multiplex PCR did not identify species, and further testing was not performed.

### Regional variations in AMR

Macrolide (*n* = 70/91, 76.9%) and lincosamide (*n* = 50/61, 81.9%) resistance were found mainly in *Campylobacter* from Québec (Supplementary Table S5). Tetracycline-resistant *Campylobacter* was isolated frequently from Ontario (*n* = 192/458, 41.9%) and the Western region (*n* = 192/458, 41.9%), whereas fluoroquinolone-resistant *Campylobacter* was isolated mostly from the Western region (*n* = 215/300, 71.7%). *Campylobacter* resistance to ketolides was found in Ontario only. The MDR was observed in *Campylobacter* isolates from Ontario (*n* = 14/17, 82.4%) and Québec (*n* = 3/17, 17.6%) (Supplementary Table S5).

### Analysis of AMR patterns and clustering

The most commonly observed AMR patterns in *Campylobacter* isolates included ciprofloxacin-nalidixic acid-tetracycline (Table 2). Multidrug resistance (MDR) (≥3 antimicrobial classes) among isolates was low (17/1063, 1.60% (95% CI: 0.96–2.60%). Of the 17 MDR isolates, 8 were *C.* spp (10.53%; 95% CI: 4.98–20.21%), 6 were *C. coli* (1.79%; 95% CI:0.73–4.04%), and 3 were *C. jejuni* (0.46%; 95% CI:0.12–1.46%). The most common MDR pattern observed was azithromycin-clindamycin- erythromycin-tetracyclines and azithromycin-ciprofloxacin-erythromycin-nalidixic acid-tetracyclines (Table 2).

**Table 2.** Antimicrobial resistance patterns in *Campylobacter* species isolates of Canadian turkey flocks

Antimicrobial resistance patterns	*Campylobacter n* = 1063 (%)	*Campylobacter jejuni n* = 651 (%)	*Campylobacter coli n* = 336 (%)	*C.* spp. *n* = 76 (%)
TET	280	218 (33.5%)	52 (15.5%)	10 (13.2%)
CIP-NAL-TET	165	102 (15.7%)	54 (16.1%)	9 (11.8%)
CIP-NAL	122	44 (6.8%)	67 (19.9%)	11 (14.5%)
AZM-CLIN-ERY	51	1 (0.2%)	46 (13.7%)	4 (5.3%)
AZM-ERY	20	1 (0.2%)	19 (5.7%)	–
**AZM-CLIN-ERY-TET** ^a^	**4**	**3 (0.5%)**	**1 (0.3%)**	–
**AZM-CIP-ERY-NAL-TET**	**4**	–	**1 (0.3%)**	**3 (3.9%)**
**AZM-CIP-CLIN-ERY-NAL-TET**	**3**	–	**2 (0.6%)**	**1 (1.3%)**
AZM-CIP-ERY-NAL	3	–	3 (0.9%)	–
**AZM-ERY-TEL-TET**	**3**	–	**2 (0.6%)**	**1 (1.3%)**
**AZM-CIP-CLIN-ERY-NAL**	**1**	–	–	**1 (1.3%)**
**AZM-CIP-CLIN-ERY-NAL-TEL**	**1**	–	–	**1 (1.3%)**
**AZM-CLIN-ERY-TEL-TET**	**1**	–	–	**1 (1.3%)**
CIP-TET	1	–	1 (0.3%)	–

a Multidrug-resistant patterns (resistance ≥3 classes) are highlighted in light grey colour.

**Figure 1.**
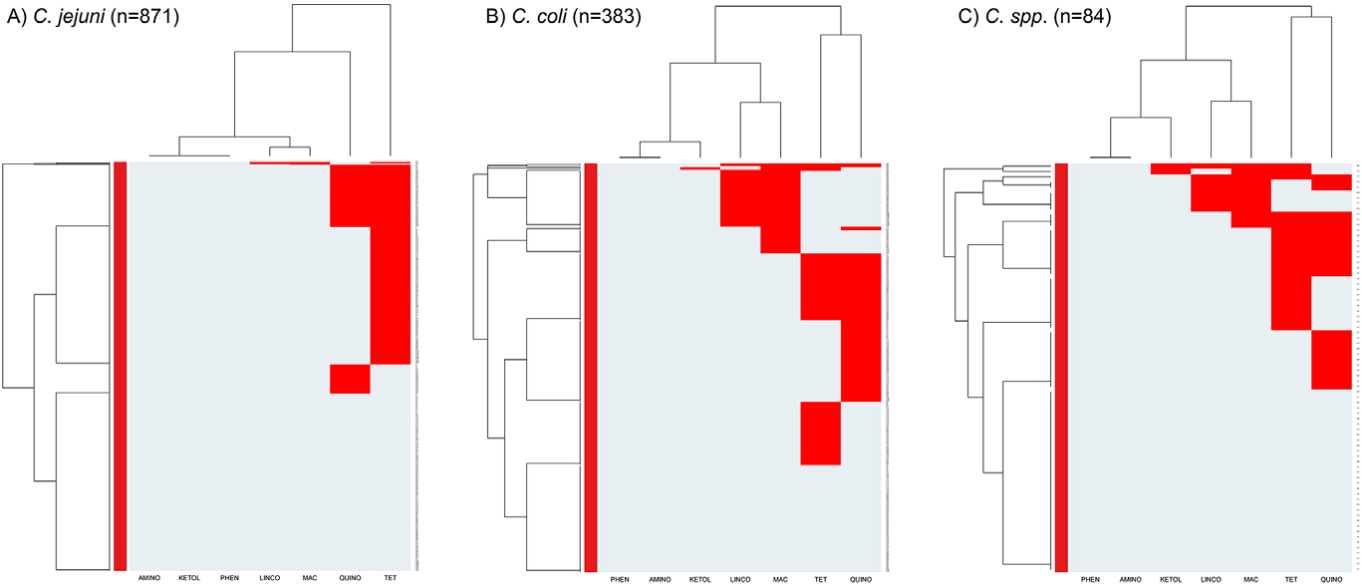
Heatmap of antimicrobial resistance patterns in *Campylobacter jejuni* (a), *Campylobacter coli* (b), *and C.* spp. (c) isolates collected from Canadian turkey flocks during 2016–2021. *X*-axes represent the antimicrobial classes: Aminoglycosides (AMINO), Ketolides (KETOL), Lincosamides (LINCO), Phenicols (PHEN), Macrolides (MAC), Fluoroquinolones (QUINO), and Tetracyclines (TET). *Y*-axes represent the *Campylobacter* isolates included in this study. The red colour depicts the resistant patterns.

The heatmaps with clustering dendrograms Figure 1 represent AMR by antimicrobial class (columns) among the *Campylobacter* species isolates (rows). Two clusters of AMR patterns were found in the *C. jejuni* heatmap (Figure 1a). The first cluster included resistance to tetracyclines only. The second AMR cluster had two subclusters: a cluster of fluoroquinolone resistance and a cluster that included resistance to macrolides, lincosamides, and susceptibility to aminoglycosides and phenicols. The heatmap of *C. coli* and *Campylobacter* spp. (Figure 1b,c) also showed two main clusters. One is a cluster of resistance to fluoroquinolones and tetracycline. The other cluster contains two AMR subclusters: one macrolide and lincosamide-resistant cluster, and one cluster of ketolide resistance and aminoglycoside and phenicol susceptibility. The rows of heatmaps (Figure 1) showed a cluster of isolates resistant to ≥3 antimicrobial classes and a second cluster of isolates with subclusters of susceptibility to all antimicrobial classes and resistance to one or two antimicrobial classes.

**Figure 2.**
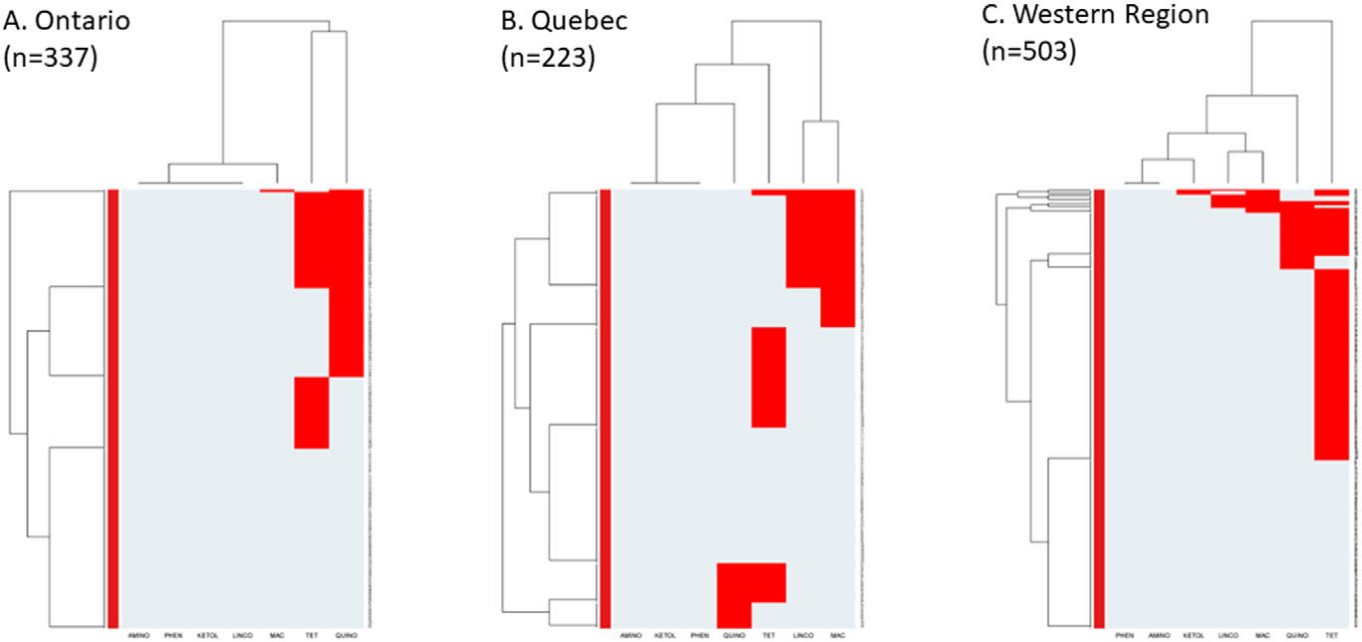
Heatmap of antimicrobial resistance by region in *Campylobacter* isolates collected from Canadian turkey flocks during 2016–2021. Ontario (a), Quebec (b), and Western region (c). *X*-axes represent the antimicrobial classes: Aminoglycosides (AMINO), Ketolides (KETOL), Lincosamides (LINCO), Phenicols (PHEN), Macrolides (MAC), Fluoroquinolones (QUINO), and Tetracyclines (TET). *Y*-axes represent the *Campylobacter* isolates included in this study. The red colour depicts the resistance patterns.

Distinct AMR patterns (columns) with two main clusters in all regions were identified in (Figure 2). Ontario (Figure 2a) had a first cluster of resistance to tetracyclines only, and a second cluster that included resistance to fluoroquinolones, macrolides, lincosamides, and ketolides. Québec (Figure 2b) contained one cluster of resistance to macrolides and lincosamides and another cluster of resistance to tetracyclines and fluoroquinolones. In contrast, the western region (Figure 2c) had two distinct clusters: one of resistance to fluoroquinolones and tetracyclines and the second of resistance to macrolides only.

### Logistic regression analysis

#### *Assessing differences in AMR between* Campylobacter *species*

**Figure 3.**
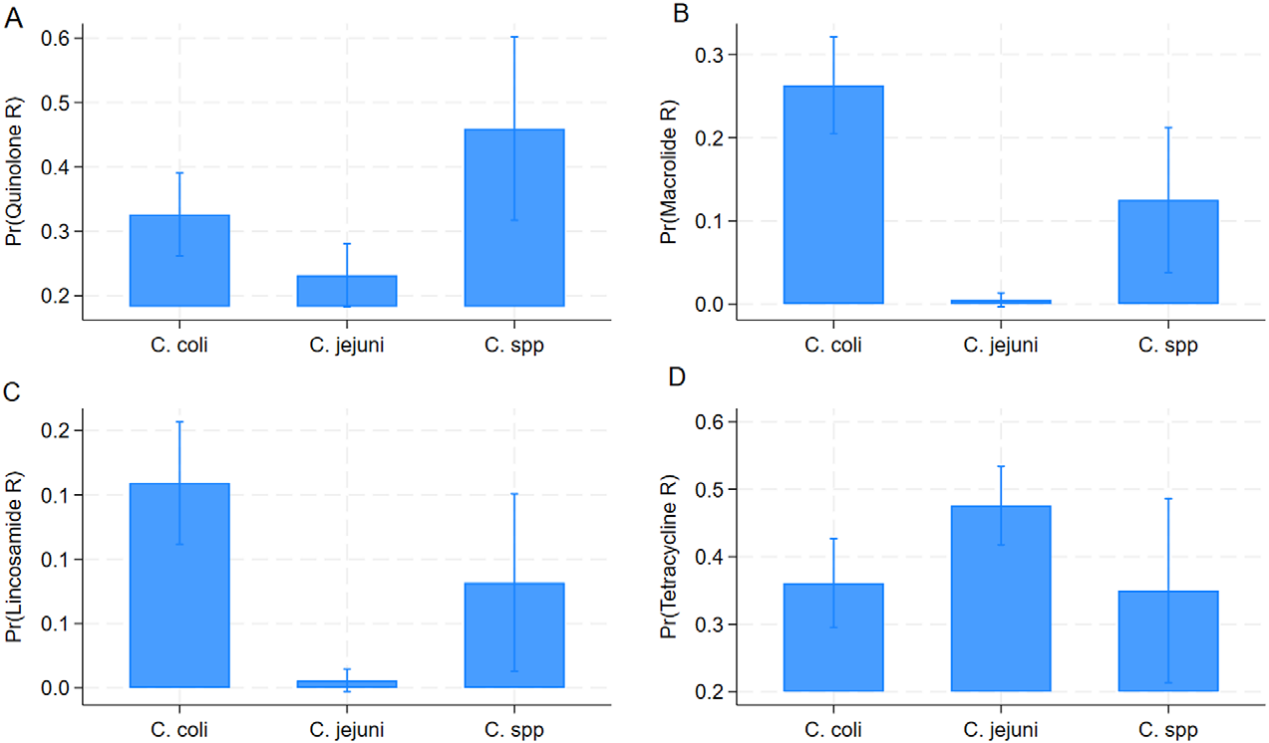
Predicted probabilities for resistance to antimicrobial classes for *Campylobacter coli*, *Campylobacter jejuni*, and *C.* spp. isolates obtained from logistic regression models using the GEE method. Fluoroquinolones (a), macrolides (b), lincosamides (c), and tetracyclines (d).

Table 3 and (Figure 3) describe the results of the regression models comparing AMR among *Campylobacter* species. The probability of *C. jejuni* isolates being resistant to fluoroquinolones (OR = 0.622) macrolides (OR = 0.014), and lincosamides (OR = 0.030) was significantly lower when compared to *C. coli.* On the other hand, *C. jejuni* isolates had a significantly higher chance of being resistant to tetracyclines (OR = 1.604) than *C. coli* isolates.

**Table 3.** Results of the logistic regression models determining associations between antimicrobial resistance and *Campylobacter* species (*n* = 1063)

Antimicrobial resistance^a^	Campylobacter species	Odds ratio (95% CI)	*p*-values
Macrolides	*Campylobacter coli*	Referent	–
	*Campylobacter jejuni*	0.01 (0.00–0.062)	<0.001
	*C.* spp.	0.40 (0.18–0.88)	0.02
	Intercept	0.36 (0.26–0.48)	<0.001
Fluoroquinolones	*C. coli*	Referent	–
	*C. jejuni*	0.62 (0.47–0.83)	0.001
	*C.* spp.	1.75 (0.96–3.21)	0.07
	Intercept	0.48 (0.36–0.65)	<0.001
Lincosamides	*C. coli*	Referent	–
	*C. jejuni*	0.03 (0.01–0.12)	<0.001
	*C.* spp.	0.47 (0.19–1.15)	0.10
	Intercept	0.19 (0.13–0.27)	<0.001
Tetracyclines	*C. coli*	Referent	–
	*C. jejuni*	1.60 (1.24–2.08)	<0.001
	*C.* spp.	0.95 (0.51–1.78)	0.88
	Intercept	0.57 (0.43–0.75)	<0.001

a Antimicrobial resistance found in ≥5% and ≤95% isolates were only included for this study.

#### Assessing differences in AMR between regions

**Figure 4.**
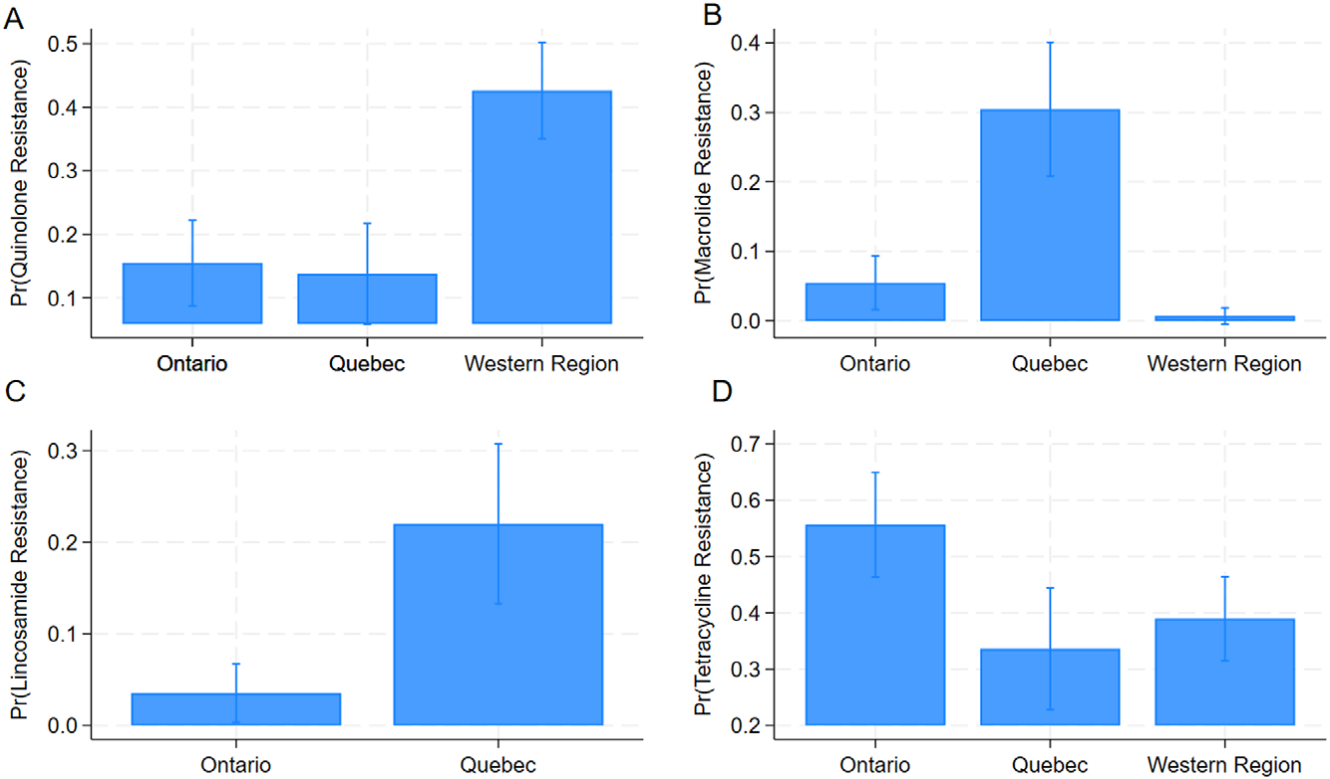
Predicted probabilities for resistance to antimicrobial classes in *Campylobacter* isolates among regions obtained from logistic regression models using the GEE method. Fluoroquinolones (a), macrolides (b), lincosamides (c), and tetracyclines (d).

Table 4 and (Figure 4) describe the results of the logistic regression models on the regional differences in AMR among *Campylobacter* isolates.

**Table 4.** Results of logistic regression models evaluating associations between antimicrobial resistance to antimicrobial classes and Canadian regions

Antimicrobial resistance^a^	Regions	Odds ratio (95% CI)	*p*-values
Macrolides	Ontario	Referent	–
	Quebec	7.59 (3.16–18.24)	<0.001
	Western	0.12 (0.02–0.77)	0.03
	Intercept	0.06 (0.03–0.12)	<0.001
Fluoroquinolones	Ontario	Referent	–
	Quebec	0.87 (0.38–2.03)	0.75
	Western	4.06 (2.22–7.41)	<0.001
	Intercept	0.18 (0.11–0.31)	<0.001
Lincosamides	Ontario	Referent	–
	Quebec	7.71 (2.67–22.28)	<0.001
	Western	1.00	NA
	Intercept	0.19 (0.13–0.27)	<0.001
Tetracyclines	Ontario	Referent	–
	Quebec	0.40 (0.22–0.75)	0.01
	Western	0.51 (0.31–0.83)	0.01
	Intercept	1.25 (0.86–1.83)	0.27

a Antimicrobial resistance found in ≥5% and ≤95% isolates were only included for this study.

*Campylobacter* isolates from the Western region had a significantly higher chance of being resistant to fluoroquinolones (OR:4.06; 95% CI: 2.22–7.41) than isolates from Ontario. The probability of an isolate being resistant to macrolides (OR:7.59; 95% CI: 3.16–15.24) and lincosamides (OR: 7.71; 95% CI: 2.67–22.28) was significantly higher if they originated from Quebec compared to Ontario. Isolates from Ontario had significantly higher odds of being resistant to tetracyclines than isolates from Quebec (OR:0.40; 95% CI: 0.22–0.75) and the Western region (OR:0.51; 95% CI: 0.31–0.83).

#### Assessing associations between AMR and AMU

**Figure 5.**
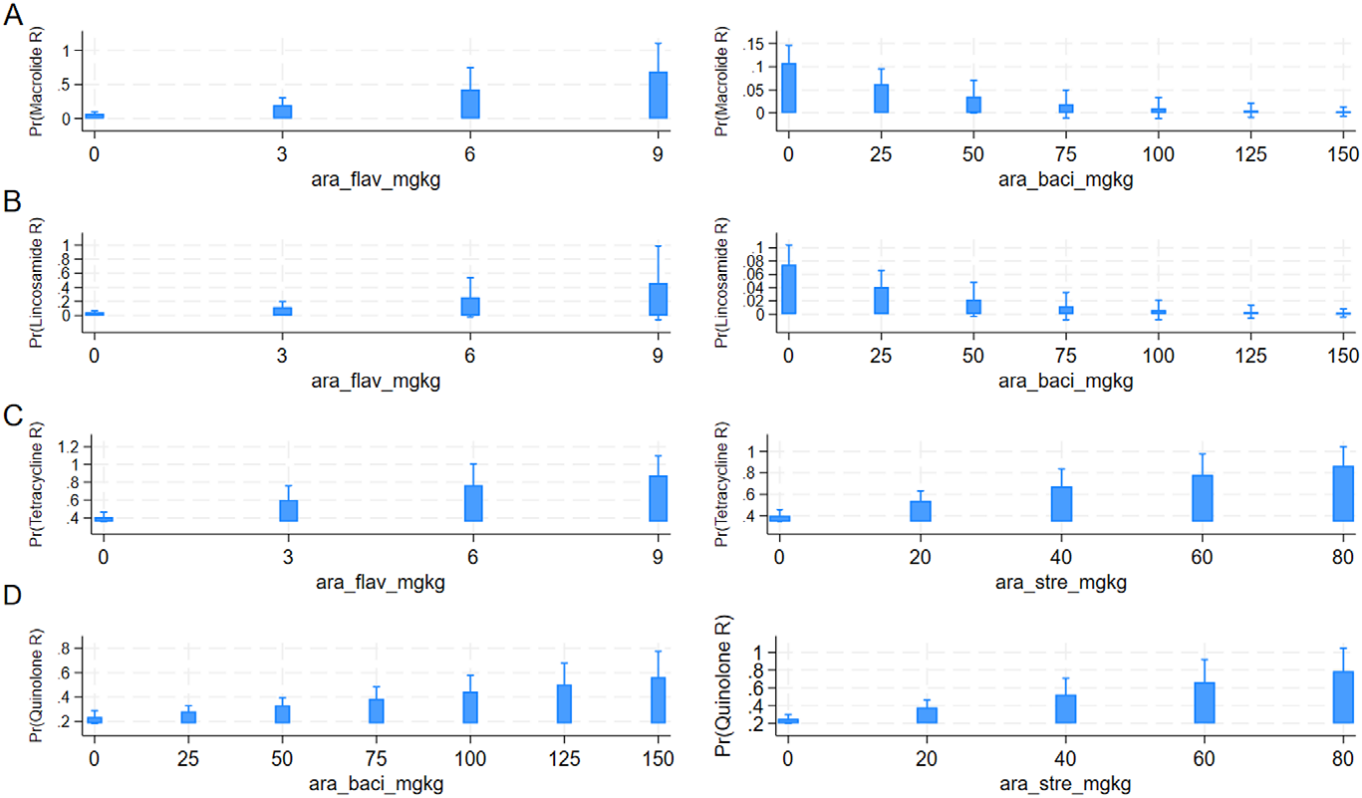
Predicted probabilities for resistance to antimicrobial classes and antimicrobial use indicators obtained from the multivariable logistic regression models using GEE methods. Outcomes: Resistance to macrolides (a), lincosamides (b), tetracyclines (c), and fluoroquinolones (d). Predictors: Use of flavophospholipids (FLAV), bacitracins (BACI), and streptogramins (STRE).

Bacitracin (*n* = 94 flocks), aminoglycosides (*n* = 82), streptogramins (*n* = 56), flavophospholipids (*n* = 36), beta-lactams (*n* = 35), orthomycins (*n* = 22) and trimethoprim-sulfamethoxazole (*n* = 16) were used in ≥5% of 293 flocks (Table 5 and Supplementary Table S6) therefore these antimicrobials were included as predictor variables in the AMR-AMU GEE models. Multivariable model results showed that fluoroquinolone resistance was associated with the use of bacitracin (OR: 1.01; 95% CI: 1.002–1.02) and streptogramins (OR: 1.03; 95% CI: 1.01–1.05); however, it should be noted that the effect estimates were small. Resistance to tetracyclines was strongly associated with the use of flavophospholipids (OR: 1.27; 95% CI: 1.01–1.60) and moderately with streptogramins (OR: 1.03; 95% CI: 1.01–1.05). Similarly, the use of flavophospholipids (OR: 1.53; 95% CI: 1.17–1.99) was strongly associated with macrolide resistance however the use of bacitracin was protective (OR: 0.98; 95% CI: 0.95–0.99) for macrolide resistance. Similar associations were observed for lincosamide resistance in *Campylobacter.* The use of flavophospholipids (OR: 1.40; 95% CI: 1.05–1.87) was strongly associated with lincosamide resistance, whereas the use of bacitracin (OR: 0.97; 95% CI: 0.95–0.99) was protective for lincosamide resistance. The predicted probabilities for resistances to individual antimicrobial classes and AMU obtained from the multivariable logistic regression models are presented in Figure 5.

**Table 5.** Quantity of antimicrobials used in turkey farms where *Campylobacter* was isolated by sampling year

Antimicrobials	Year	Number of farms	Quantity (mg/kg biomass)			
			Total	Mean (SD)	Min	Max
Aminoglycosides	2016	40	9.45	2.57 (0.49)	0.24	0.06
	2017	34	5.59	1.8 (0.29)	0.16	0.06
	2018	7	9.15	7.04 (2.58)	1.31	0.03
	2019	1	33.44	33.44 (−)	33.44	33.44
	2020	0	0	0 (0)	0	0
	2021	0	0	0 (0)	0	0
Macrolides	2016	5	115.19	4.88 (−)	28.8	41.09
	2017	4	41.07	1.4 (−)	13.69	30.57
	2018	0	0	0 (−)	0	0
	2019	0	0	0 (−)	0	0
	2020	0	0	0 (−)	0	0
	2021	0	0	0 (−)	0	0
Beta-lactams	2016	9	32.56	8.57 (3.5)	3.62	0.04
	2017	4	8.02	4.2 (1.74)	2	0.02
	2018	8	73.71	34.7 (11.67)	9.21	0.05
	2019	4	92.16	29.73 (5.6)	23.04	16.46
	2020	1	18.09	18.09 (−)	18.09	18.09
	2021	5	218.18	102.77 (44.57)	43.64	1.49
Trimethoprim-sulfamethoxazole	2016	4	129.38	47.79 (15.31)	32.34	15.26
	2017	5	416.33	173.35 (55.63)	83.27	27.81
	2018	1	149.61	149.61 (−)	149.61	149.61
	2019	4	322.52	133.47 (50.67)	80.63	20.34
	2020	2	145.92	89.79 (23.8)	72.96	56.14
	2021	0	0	0 (0)	0	0
Bacitracin	2016	16	1012.50	114.3 (21.9)	63.28	35.45
	2017	16	995.78	104.83 (28.08)	62.24	15.73
	2018	13	1004.64	123.5 (33.94)	77.28	0.95
	2019	39	2613.7	132.67 (26.32)	67.02	26.77
	2020	4	132.7	48.51 (13.66)	33.18	17.69
	2021	7	472.73	108.13 (29.11)	67.53	32.59
Tetracycline	2016	4	212.74	71.65 (34.93)	53.19	0.83
	2017	1	3.19	3.19 (−)	3.19	3.19
	2018	3	8.04	7.04 (3.78)	2.68	0.4
	2019	2	54.42	48.8 (30.54)	27.21	5.61
	2020	1	1	1 (−)	1	1
	2021	1	58.80	58.8 (−)	58.8	58.8
Flavophospholipids	2016	2	1.01	2.91 (3.81)	1.9	0.89
	2017	9	1.42	6.36 (26.72)	2.97	1.8
	2018	2	0.43	3.12 (5.37)	2.68	2.25
	2019	2	1.69	3.8 (4.22)	2.11	0.42
	2020	7	0.89	5 (25.45)	3.64	2.13
	2021	14	1.01	3.97 (24.82)	1.77	0.56
Orthomycins	2016	0	0	0 (0)	0	0
	2017	0	0	0 (0)	0	0
	2018	0	0	0 (0)	0	0
	2019	3	31.55	12.78 (2)	10.52	8.97
	2020	5	95.18	22.9 (4.01)	19.04	13.11
	2021	14	225.75	35.85 (10.17)	16.12	1.5
Streptogramins	2016	17	424.72	42.42 (11.22)	24.98	10.45
	2017	15	381.89	62.01 (13.21)	25.46	8.22
	2018	21	524.51	42.8 (10.39)	24.98	1.53
	2019	3	45.51	27.49 (10.91)	15.17	6.72
	2020	0	0	0 (0)	0	0
	2021	0	0	0 (0)	0	0

The use of flavophospholipids in turkey flocks was significantly associated with the selection of MDR isolates (OR: 2.32; 95% CI: 1.56–3.45).

## Discussion

The emergence and persistence of AMR in *Campylobacter* isolated from poultry pose a food safety risk due to the limited treatment availability for severe human campylobacteriosis. This study evaluated various AMR outcomes in *C. jejuni*, *C. coli,* and other *Campylobacter* (un-speciated) isolates of turkey farm samples and relate the findings with the AMU data collected between 2016 and 2021. *Campylobacter* isolates exhibited phenotypic resistance to WHO’s Highest Priority-Critically Important Antimicrobials (HP-CIAs: fluoroquinolones, macrolides, and ketolides) and highly important antimicrobials (HIA’s: tetracyclines and lincosamides) but only a few isolates were deemed MDR. The AMR patterns of isolates differed between species (*C. jejuni* vs. *C. coli*) and among Canadian regions. Associations between the resistance and use of antimicrobials from the same antimicrobial class could not be examined due to the very low use of these antimicrobials (e.g., tetracyclines and fluoroquinolones) during the study period (<5%), but associations were detected between resistance to certain antimicrobial classes (e.g., fluoroquinolone) and the use of unrelated antimicrobial classes (e.g., bacitracin and streptogramins). The analysis indicated high resistance to the tetracycline and fluoroquinolone classes in *Campylobacter*; however, there was limited use of these antimicrobials during the study period. Future studies should investigate whether non-AMU factors (e.g., the use of cleaning products) might impact the selection of AMR in *Campylobacter.*

The high prevalence of tetracycline resistance in the *Campylobacter* isolates observed in this study agrees with the results of previous studies from Canada, Europe, Asia, and other countries [6–8, 10, 11, 13–16, 28]. Tetracycline-resistant *Campylobacter* isolates were also reported in retail meats, poultry flocks, the environment, and humans [6, 10, 29, 30]. A plasmid-encoded gene tet(O) was found to be responsible for the tetracycline resistance in *Campylobacter* in previous studies [29, 31], and this gene was reported to be horizontally transferred between *C. jejuni* and *C. coli* in the gut of food animals [30, 32]. This study found that nearly half (49%) of the *C. jejuni* isolates and one-third (33%) of *C. coli* isolates were resistant to tetracyclines. The findings of tetracycline-resistant isolates could be due to co-selection for resistance [33] or the historical use of tetracyclines for treatment in turkeys. In our study, the use of tetracyclines was seldom reported, suggesting historical tetracyclines use may have resulted in the persistence of tetracycline-resistant *Campylobacter* isolates in Canadian turkey flocks. Nonetheless, findings of tetracycline resistance in *C. jejuni* and *C. coli* isolates should not be ignored as both species are known to cause disease in humans, and there is the potential for transmission to people through turkey meat which is a popular food choice in Canada [34]. In light of this, it is vital to continue ongoing AMU and AMR surveillance in turkey flocks. These findings should be integrated with findings from studies examining AMR in humans, slaughter plants, industrial food processing facilities, and the environment and be complemented by examining biosecurity practices, particularly those practices such as cleaning, disinfection, and downtime that may lead to carry-over of resistant organisms from flock to flock [20].

Fluoroquinolone resistance in *Campylobacter* isolates is one of the major concerns worldwide because of limited treatment options for infections with drug-resistant isolates [4]. *Campylobacter* resistance to fluoroquinolones was previously detected in chickens, turkeys, humans, and environments in Canada and other countries. In line with previous studies, our study also identified a higher proportion of fluoroquinolone resistance in *C. coli* (38.1%) than in *C. jejuni* (22.4%) [7, 8, 12]. Associations between fluoroquinolone use in poultry and increased fluoroquinolone resistance in *Campylobacter* isolates have been described previously; however, some studies have also reported a high proportion of fluoroquinolone-resistant *Campylobacter* isolates in the absence of fluoroquinolone use, which agrees with our study’s finding [7, 11, 12]. This finding might be explained by past fluoroquinolone use in Canadian turkey flocks that may have caused the preservation of fluoroquinolone-resistant *Campylobacter* isolates in the farm environment.

It was previously described that a point mutation in the gyrA gene at the Thr-86 position leads to fluoroquinolone resistance in the *Campylobacter* isolates and provides favourable conditions for the pathogen to colonize the turkeys’ gut without AMU selection pressure [12, 35]. In European countries, the clonal spread of fluoroquinolone-resistant *Campylobacter* isolates was observed [7]. A comprehensive Australian study evaluated the AMR determinants of *C. coli* and *C. jejuni* that were isolated from chicken cecal samples at slaughter. The genomic characteristics of fluoroquinolone-resistant isolates identified humans, pest species, or wild birds as the most likely source of these isolates [11], indicative of potential reverse-zoonosis events and inter-species movement of resistant *Campylobacter* [31]. Nevertheless, AMU and fluoroquinolone resistance mechanisms in *Campylobacter* from turkeys still require further investigation including the incorporation of genomics. The monitoring of MIC shifts indicative of the mutation presences could be important for the detection of high-level resistance [36]. Ultimately, fluoroquinolone-resistant *Campylobacter* is a concern as it may lead to treatment failure and symptomatic relapse if people consume contaminated, undercooked or improperly handled turkey products.

Macrolides are the first choice of antibiotics to treat human campylobacteriosis [37]. In studies conducted in Canada and other countries, a low prevalence of macrolides-resistant *Campylobacter* was reported in turkey flocks and turkey meat [6, 7, 10, 12, 13]. Several studies have also reported low resistance to lincosamides in *Campylobacter* isolates [12, 13], which coincides with this study’s results. We found that *C. coli* had a higher prevalence of resistance to macrolides and lincosamides than *C. jejuni.* Mutations in the 23S rRNA gene (rrnB operon) or modifications of the ribosomal proteins (L4 and L22) or CmeABC efflux pump activity or a combination of these mechanisms can result in the development of macrolide-resistant *Campylobacter* [38]. In chickens, it was shown that the substitutions in the 23S rRNA gene (A2075G or A2074C/G) reduced the colonization of flocks with *C. jejuni* [38]. We presume the same mechanism occurred in our study as we found a few macrolide-resistant *C. jejuni.* Further investigation using molecular techniques is required to confirm this theory.

We found that all isolates were susceptible to aminoglycosides and phenicols, and only two isolates were resistant to ketolides, which is in agreement with other previous studies conducted in Canada and the European Union [6, 8, 12] that reported a very low level of resistance.

The presence of multidrug resistance in *Campylobacter* isolates reported in several previous studies is high compared to our study [9, 10, 28, 30]. We found a very low frequency of MDR isolates and a low number of isolates had co-resistance to the macrolides-lincosamides-tetracyclines (MLT) classes or the macrolides-fluoroquinolones-tetracyclines (MFT) classes. The co-resistance of tetracyclines with macrolides-lincosamides is of concern due to their genetic linkages on transposons [39]. These linkages could recombine, develop a novel chimeric element, and allow for co-selection which could be transferred readily among Gram-positive and Gram-negative bacteria [39]. This could lead to limited treatment options and could also result in therapeutic failure and severe consequences [36]. Fortunately, we found that MDR in *Campylobacter* isolates from Canadian turkey flocks has remained low, which is a promising finding from a public health perspective.

The clustering analysis in our study demonstrated a difference in the concurrent resistance patterns with tetracycline-resistant *C. jejuni* and fluoroquinolone-tetracycline-resistant *C. coli* or other *Campylobacter species.* Our analysis also indicated a concurrent resistance to macrolides and lincosamides in all the species. Similarly, there were distinct resistance patterns among the three regions of Canada. The Western region had a fluoroquinolone-tetracycline-resistant cluster without MDR isolates, while Quebéc had a macrolide-lincosamide-resistant cluster, whereas Ontario had a tetracycline-resistant cluster. Differences in the AMR trends between species and regions warrant future studies to identify other factors besides AMU that might drive the emergence of resistance. These findings emphasize the need for ongoing monitoring of turkey farms to identify risk factors for AMR development and assess the prudent use of commonly used antimicrobials to help mitigate the occurrences of AMR in turkey flocks and the food chain.

Our logistic regression analysis showed that fluoroquinolone and macrolide resistance was more likely to be detected in *C. coli* isolates than in *C. jejuni.* Tetracycline resistance was more likely observed in *C. jejuni* isolates than in *C. coli.* These findings could be partly explained by the presence or transfer of resistance genes among different *Campylobacter* species or other factors such as the presence of other animals or birds that can transfer drug-resistant *Campylobacter* to turkeys. The underlying reasons for the difference in the resistance between various *Campylobacter* species require further investigation. Such investigations could include examining if there is an age-related shift in the composition of *Campylobacter* populations, or if bird breed, management, or production (organic, conventional, or antibiotic-free) factors impact AMR.

AMU is a well-known attribution factor for developing AMR in commensal and pathogenic enteric bacteria, including *Campylobacter* [7, 21]. It is hypothesized that any AMU may have favoured the colonization of turkey flocks with drug-resistant-*Campylobacter* due to the selective pressure of AMU. However, our regression analysis using GEE methods only indicated associations between resistance to tetracyclines and fluoroquinolones in the *Campylobacter* isolates and the use of unrelated antimicrobials, particularly bacitracin, and streptogramins. We found an association between the resistance to tetracyclines (OR: 1.27; 95% CI: 1.01–1.60), macrolides (OR: 1.53; 95% CI: 1.17–1.99), and lincosamides (OR: 1.40; 95% CI: 1.05–1.87) and the use of flavophospholipids in the turkey flocks. Moreover, the odds of an isolate being MDR were associated with the use of flavophospholipids on turkey farms. Flavophospholipids (flavomycin or bamermycin) are mainly used in poultry as feed additives, which were reported to have potential plasmid-curing activity [40, 41]. However, this feed additive was found to promote the *mefA* genes responsible for macrolide resistance in *Enterococcus* species [42]. Although flavophospholipids are beneficial, the alteration in the turkey gut microflora may have favoured the selection of AMR in *Campylobacter* isolates. Nevertheless, comprehensive molecular epidemiological investigations are warranted to confirm the statistical findings and to investigate the role of AMU, particularly flavophosphoolipids, in the development of AMR in *Campylobacter* species.

Before extrapolating our study’s results some limitations need to be noted. Turkey flocks were visited only once at the end of their growth out period. Future studies should sample turkey flocks throughout their life to identify age-dependent AMU-AMR factors.

In conclusion, the present study identified a high proportion of *C. jejuni* and *C. coli* isolates that were resistant to tetracyclines and fluoroquinolones, despite the very low use of these antimicrobials in the studied turkey flocks. Macrolide and lincosamide resistances were found to be higher in *C. coli* and other *Campylobacter* species compared to *C. jejuni*, suggesting that antimicrobial-resistant *C. coli* might be better adapted to surviving in the turkey farm environment and colonizing turkeys more easily than *C. jejuni* [43]. The very low to null prevalence of resistance to aminoglycosides, phenicols, and ketolides and the low occurrence of MDR *Campylobacter* in Canadian turkey flocks is an encouraging finding for public health. This study also provides a basis for the longitudinal monitoring of AMU and phenotypic AMR in *Campylobacter* isolates from Canadian turkey flocks that may aid in future surveillance activities. However, the inclusion of molecular epidemiological studies is needed to better understand the persistence of resistant isolates in turkey flocks in Canada.

## Supporting information

Shrestha et al. supplementary material Shrestha et al. supplementary material

## Data Availability

All materials needed to replicate the findings of the article are available upon request.

## Supplementary material

For supplementary material accompanying this paper visit http://doi.org/10.1017/S0950268823001462. click here to view supplementary material
