# Supplementary material for: Assessing antimicrobial resistance in Campylobacter jejuni and Campylobacter coli and its association with antimicrobial use in Canadian turkey flocks
Source: Epidemiol Infect. 2023 Sep 5;151:e152. doi: 10.1017/S0950268823001462 (PMC10548540; doi:10.1017/S0950268823001462)
Supplement: Shrestha et al. supplementary material [file S0950268823001462sup001.docx]

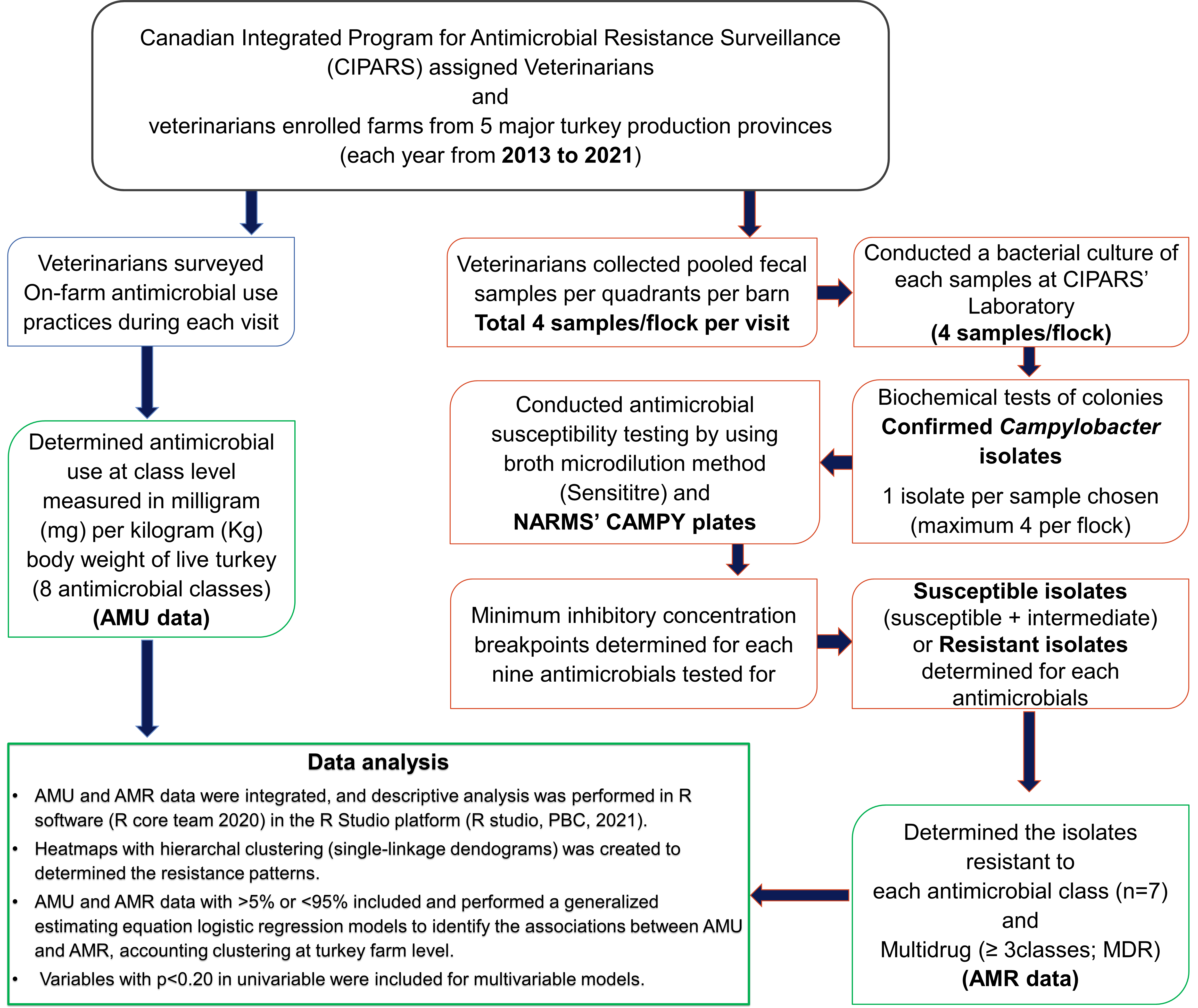


**Fig S1: Diagram of the study design including sample and AMU data collection, *Campylobacter* isolation, AMR identification, and data analysis**


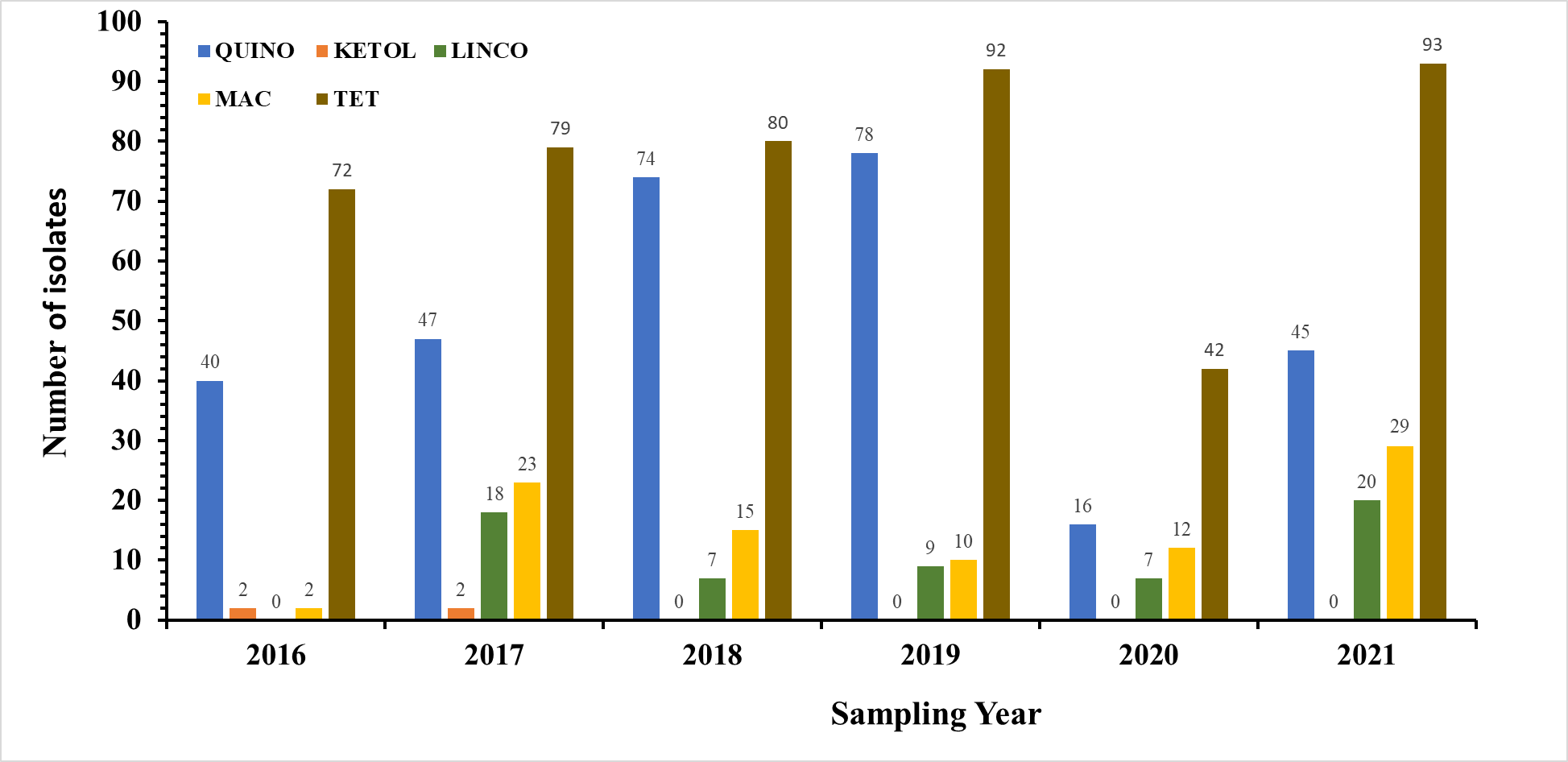


**Fig S2: Number of *Campylobacter* isolates resistant to antimicrobial classes by sampling year:** Fluoroquinolones (QUINO), Ketolides (KETOL), Lincosamides (LINCO), Macrolides (MAC), Tetracyclines (TET)

**Table S1: References for previous studies or websites that explain a detailed method of CIPARS farm surveillance** [1–5].

| **References:** |
| --- |
| 1. A. Agunos, S. P. Gow, D. F. Léger, A. E. Deckert, C. A. Carson, A. L. Bosman, S. Kadykalo, & R. J. Reid-Smith, Antimicrobial Use Indices—The Value of Reporting Antimicrobial Use in Multiple Ways Using Data From Canadian Broiler Chicken and Turkey Farms. *Frontiers in Veterinary Science*, **7** (2020). https://doi.org/10.3389/fvets.2020.567872.  2. A. Agunos, S. P. Gow, A. E. Deckert, G. Kuiper, & D. F. Léger, Informing Stewardship Measures in Canadian Food Animal Species through Integrated Reporting of Antimicrobial Use and Antimicrobial Resistance Surveillance Data&mdash;Part I, Methodology Development. *Pathogens*, **10** (2021). https://doi.org/10.3390/pathogens10111492.  3. A. Agunos, S. P. Gow, A. E. Deckert, & D. F. Léger, Informing Stewardship Measures in Canadian Food Animal Species through Integrated Reporting of Antimicrobial Use and Antimicrobial Resistance Surveillance Data—Part II, Application. *Pathogens*, **10** (2021) 1491. https://doi.org/10.3390/pathogens10111491.  4. R. D. Shrestha, A. Agunos, S. P. Gow, A. E. Deckert, & C. Varga, Associations between antimicrobial resistance in fecal Escherichia coli isolates and antimicrobial use in Canadian turkey flocks, 2016-2019. *Frontiers in Microbiology*, (n.d.) 2902.  5. L. Huber, A. Agunos, S. P. Gow, C. A. Carson, & T. P. Van Boeckel, Reduction in antimicrobial use and resistance to Salmonella, Campylobacter, and Escherichia coli in broiler chickens, Canada, 2013–2019. *Emerging Infectious Diseases*, **27** (2021) 2434. |

**Table S2: Antimicrobial agents by antimicrobial classes that were included in the AMR and AMU analysis.**

| Resistance to Antimicrobial Class **(Antimicrobial agents)** | **Antimicrobials used in turkey flocks at class level (Antimicrobial agents)** |
| --- | --- |
| Aminoglycosides (Gentamicin) | Aminoglycosides (Gentamicin) |
| Ketolides (Telithromycin) | Bacitracins (Bacitracins) |
| Lincosamides (Clindamycin) | Beta-lactams (Penicillins) |
| Macrolides (Azithromycin & Erythromycin) | Macrolides (Azithromycin & Erythromycin) |
| Phenicols (Florfenicol) | Quinolones (Ciprofloxacin & Nalidixic acid) |
| Quinolones (Ciprofloxacin & Nalidixic acid) | Streptogramins (Virginiamycin) |
| Tetracyclines (Tetracycline) | Trimethoprim-sulfamethoxazole |
|  | Orthomycins |
|  | Flavophospholipids (Flavinomycins) |
|  | Tetracyclines (Tetracycline) |

**Table S3: Total number of farms sampled, and *Campylobacter* isolated by year**

| **Sampling**  **year** | **Number of turkey farms enrolled and sampled** | Number of | | | |
| --- | --- | --- | --- | --- | --- |
|  |  | ***Campylobacter*** | ***C. jejuni*** | ***C. coli*** | ***C. spp.*** |
| 2016 | 47 | 171 | 107 | 64 | 0 |
| 2017 | 45 | 157 | 60 | 32 | 65 |
| 2018 | 50 | 191 | 118 | 73 | 0 |
| 2019 | 58 | 214 | 145 | 60 | 9 |
| 2020 | 26 | 90 | 54 | 36 | 0 |
| 2021 | 67 | 240 | 167 | 71 | 2 |

**Table S4: Number of *Campylobacter* isolates by minimum inhibition concentration (mg/L) values or clinical breakpoint.**

| **Minimum Inhibitory**  **concentrations (mg/L)** | **Antimicrobial agents in CAMPY plate** | | | | | | | | |
| --- | --- | --- | --- | --- | --- | --- | --- | --- | --- |
|  | **AZM** | **CIP** | **CLIN** | **ERY** | **FLR** | **GEN** | **NAL** | **TEL** | **TET** |
| **0.015** | 20 | - | - | - | - | - | - | - | - |
| **0.03** | 362 | 2 | 1 | - | - | - | - | - | - |
| **0.06** | 461 | 207 | 48 | 1 | - | - | - | - | 4 |
| **0.12** | 109 | 507 | 362 | 34 | 1 | - | - | 5 | 195 |
| **0.25** | 19 | 47 | 485 | 541 | 1 | 8 | - | 106 | 296 |
| **0.5** | 1 | - | 72 | 266 | 57 | 465 | - | 189 | 67 |
| **1** | - | - | 4 | 100 | 908 | 568 | - | 110 | 33 |
| **2** | - | - | 1 | 26 | 92 | 22 | - | 58 | 4 |
| **4** | - | 12 | 29 | 4 | 4 | - | 522 | 37 | 2 |
| **8** | - | 91 | 51 | - | - | - | 233 | 10 | 6 |
| **16** | - | 156 | 8 | - | - | - | 8 | 4 | 9 |
| **32** | - | 41 | 2 | 2 | - | - | 2 | - | 26 |
| **64** | - | - | - | 36 | - | - | 32 | - | 175 |
| **128** | 91 | - | - | 53 | - | - | 266 | - | 248 |
| Antimicrobial agents: AZM: azithromycin; CIP: ciprofloxacin; CLIN: clindamycin; ERY: erythromycin; FLR: florfenicol; GEN: gentamicin; NAL: Nalidixic acid; TEL: telithromycin; TET: tetracycline | | | | | | | | | |

**Table S5: Summary of the antimicrobial resistance in *Campylobacter* isolates by regions and years (2016-2021).**

| Region | Year | Resistance to Antimicrobials^a^ | | | | | | | | | | | MDR^b^ |
| --- | --- | --- | --- | --- | --- | --- | --- | --- | --- | --- | --- | --- | --- |
|  |  | **AZM** | **CHL** | **CIP** | **CLIN** | **ERY** | **FLR** | **GEN** | **MEM** | **NAL** | **TEL** | **TET** |  |
| Ontario | 2016 | 2 | 0 | 3 | 0 | 2 | 0 | 0 | 0 | 3 | 2 | 46 | 2 |
|  | 2017 | 8 | 0 | 6 | 4 | 8 | 0 | 0 | 0 | 5 | 2 | 28 | 8 |
|  | 2018 | 4 | 0 | 13 | 4 | 4 | 0 | 0 | 0 | 13 | 0 | 28 | 1 |
|  | 2019 | 1 | 0 | 13 | 1 | 1 | 0 | 0 | 0 | 13 | 0 | 22 | 0 |
|  | 2020 | 0 | 0 | 3 | 0 | 0 | 0 | 0 | 0 | 3 | 0 | 10 | 0 |
|  | 2021 | 3 | 0 | 14 | 2 | 3 | 0 | 0 | 0 | 14 | 0 | 58 | 3 |
| Quebec | 2016 | 0 | 0 | 0 | 0 | 0 | 0 | 0 | 0 | 0 | 0 | 12 | 0 |
|  | 2017 | 14 | 0 | 0 | 14 | 14 | 0 | 0 | 0 | 0 | 0 | 6 | 3 |
|  | 2018 | 9 | 0 | 2 | 3 | 9 | 0 | 0 | 0 | 2 | 0 | 7 | 0 |
|  | 2019 | 9 | 0 | 14 | 8 | 9 | 0 | 0 | 0 | 14 | 0 | 29 | 0 |
|  | 2020 | 12 | 0 | 0 | 7 | 12 | 0 | 0 | 0 | 0 | 0 | 4 | 0 |
|  | 2021 | 26 | 0 | 17 | 18 | 26 | 0 | 0 | 0 | 17 | 0 | 16 | 0 |
| Western | 2016 | 0 | 0 | 37 | 0 | 0 | 0 | 0 | 0 | 37 | 0 | 14 | 0 |
|  | 2017 | 1 | 0 | 41 | 0 | 1 | 0 | 0 | 0 | 41 | 0 | 45 | 0 |
|  | 2018 | 2 | 0 | 59 | 0 | 2 | 0 | 0 | 0 | 59 | 0 | 45 | 0 |
|  | 2019 | 0 | 0 | 51 | 0 | 0 | 0 | 0 | 0 | 50 | 0 | 41 | 0 |
|  | 2020 | 0 | 0 | 13 | 0 | 0 | 0 | 0 | 0 | 13 | 0 | 28 | 0 |
|  | 2021 | 0 | 0 | 14 | 0 | 0 | 0 | 0 | 0 | 14 | 0 | 19 | 0 |
| Grand Total | | **91** | **0** | **300** | **61** | **91** | **0** | **0** | **0** | **298** | **4** | **458** | **17** |
| ^a^ Antimicrobial resistance tested for: Azithromycin (AZM), Ciprofloxacin (CIP), Chloramphenicols (CHL), Clindamycin (CLIN), Erythromycin (ERY), Florfenicol (FLR), Gentamicin (GEN), Nalidixic acid (NAL), Telithromycin (TEL), Tetracycline (TET)  ^b^ Multidrug resistance (MDR) resistance to ≥3 antimicrobial classes | | | | | | | | | | | | | |

**Table S6: Total quantity of antimicrobials used in turkey farms (n=293) where Campylobacter were isolated, by year.**

| Year | Quantity of antimicrobials used (mg/kg biomass) | | |
| --- | --- | --- | --- |
|  | **Total** | **Mean (SD)** | **Median (Range)** |
| 2016 | 7,088.98 | 45.74 (29.73) | 22.72 (0.13-114.42) |
| 2017 | 6,533.36 | 53.99 (45.93) | 19.94 (0.07-181.36) |
| 2018 | 6,951.88 | 52.27 (40.21) | 66.26 (0.56-152.82) |
| 2019 | 11,259.12 | 69.93 (41.38) | 37.13 (3.8-153.93) |
| 2020 | 1,552.06 | 29.85 (26.95) | 46.81 (2.71-111.74) |
| 2021 | 3,236.20 | 37.63 (31.43) | 43.11 (1.5-175.27) |
| **Total** | **36,621.59** | **51.73 (39.68)** | **23.70 (0.07-181.36)** |
